# Precise time-matching in chimpanzee allogrooming does not occur after a short delay

**DOI:** 10.1371/journal.pone.0201810

**Published:** 2018-09-11

**Authors:** Steve Phelps, Wing Lon Ng, Mirco Musolesi, Yvan I. Russell

**Affiliations:** 1 Department of Informatics, King’s College London, London, United Kingdom; 2 Independent Scholar (affiliated with Bounded-Rationality Advancement in Computational Intelligence Laboratory), London, United Kingdom; 3 Department of Geography, University College London, London, United Kingdom; 4 Department of Psychology, Middlesex University, London, United Kingdom; 5 CRC Evolution of Social Behaviour, University of Göttingen, Göttingen, Germany; University of Portsmouth, UNITED KINGDOM

## Abstract

Allogrooming is a key aspect of chimpanzee sociality and many studies have investigated the role of reciprocity in a biological market. One theoretical form of reciprocity is time-matching, where payback consists of an equal duration of effort (e.g. twenty seconds of grooming repaid with twenty seconds of grooming). Here, we report a study of allogrooming in a group of twenty-six captive chimpanzees (Chester Zoo, UK), based on more than 150 hours of data. For analysis, we introduce a methodological innovation called the “Delta scale”, which unidimensionally measures the accuracy of time-matching according to the extent of delay after the cessation of grooming. Delta is positive when reciprocation occurs after any non-zero delay (e.g. A grooms B and then B grooms A after a five second break) and it is negative when reciprocation begins whilst the original grooming has not yet ceased. Using a generalized linear mixed-method, we found evidence for time-matched reciprocation. However, this was true only for immediate reciprocation (Delta less than zero). If there was a temporal break in grooming between two members of a dyad, then there was no evidence that chimpanzees were using new bouts to retroactively correct for time-matching imbalances from previous bouts. Our results have implications for some of the cognitive constraints that differentiate real-life reciprocation from abstract theoretical models. Furthermore, we suggest that some apparent patterns of time-matched reciprocity may arise merely due to the law of large numbers, and we introduce a statistical test which takes this into account when aggregating grooming durations over a window of time.

## Introduction

What is reciprocity? In the human sense, it refers to “a set of motivationally interrelated two-way gifts” (ref. [1], p. 137): the actors in a dyad are psychologically motivated to repay a gift with a commensurate gift (the second being contingent on the first). Reciprocity has long been a central topic of interest in the study of the evolution of cooperation [2], [3], [4], [5], [6], [7], [8], [9], [10], [11], [12], [13], one version, for example, being the idea of “reciprocal altruism” where the cost of providing an altruistic gift (“cost” measured as reduced reproductive output) is offset by the receipt of a later gift [14]. Among non-human primates, recorded examples of reciprocity-like patterns are numerous and well known [5], [6], [15]. However, some [7], [16], [17] have argued that contingent cooperation (e.g. where *A* grooms *B* *because* *B* groomed *A*, as required in reciprocal altruism) is an unlikely explanation for observed patterns of reciprocity—arguing instead that cooperation in the animal kingdom consists predominantly of cases of shorter-term *mutualism* (pseudo-reciprocity): where there is *no* pattern of “loss and delayed payback” and, instead, mutualism entails immediate benefits that flow to both parties [6], [16].

### Allogrooming as a primate currency

Allogrooming is one such “gift.” It refers to the physical action (usually using hands) of one animal rhythmically touching the body surface of another, giving focused attention to picking out dirt or parasites from the fur or skin [15], [18], [19], [20], [21], [22], [23], [24]. Allogrooming is an essential part of social life in many primate species [18], [25], [26], [27]. When primate *A* grooms primate *B*, it can be viewed as a service. Adopting the view of biological markets theory [28], [29], [30], the grooming service is construable as a naturally occurring economic transaction: primate *A*’s grooming effort benefits primate *B* both hygienically and hedonically and these benefits increase monotonically with the time invested in grooming [18], [26], [31], [32].

The natural currency of grooming has been identified as something that sustains many of the positive functions of primate sociality: bonding individuals [18], [26], [27], [33], [34], repairing and maintaining relationships [35], [36], reducing stress [37], [38] and promoting group cohesion [18], [26], [33]. The issue of allogrooming reciprocity has been investigated in primate societies, with an empirically verified contingency between receipt of grooming and payback [15]. Reciprocity in primates in biological markets has been found not only in grooming-for-grooming scenarios but also grooming-for-other-currency (interchange) scenarios, often occurring outside the influence of kin favouritism [5], [6], [15], [16], [28], [30], [39], [40], [41], [42], [43]. Chimpanzees are a well-studied exemplar in the study of primate grooming markets. Allogrooming is a key part of chimpanzee sociality, a behaviour embedded within the complex sociality of this species, occurring across disparate groups in the wild and in captivity, and which has been confirmed by researchers to have both hygienic and social/political benefits [24], [35], [37], [39], [40], [41], [42], [43], [44], [45], [46], [47], [48], [49], [50].

### Investigating duration and delay

Our goal in this paper was to investigate a very specific aspect of allogrooming: immediate versus delayed reciprocity. We investigated the time frame of reciprocation in a well-established socially complex group of captive chimpanzees [35], [37], [49], [50]. Theoretical analyses of reciprocal events [1] tend to model reciprocation as involving a discrete parcel *i* given as a gift and then reciprocated with a discrete parcel *j*. Importantly, however, the reciprocation needs to be an adequate amount. Therefore, we can also model quantitatively in terms of a *amount of i* and *amount of j* [1]. We used this “amount” variable in our analysis below: how much of the *amount* of grooming from *A* to *B* is later matched with an *amount* of grooming *B* to *A*. Thus, we pursued two main questions: to what extent do the chimpanzees reciprocate grooming, and, if so, over what time horizon? Regarding the first question, we estimated reciprocity by estimating the time-matching of grooming effort. Dyadic grooming in chimpanzees can be labelled as two forms [44], [48], [49]: one-way (*A* grooms *B* denoted *A* → *B*) or mutual (*A* grooms *B*, and simultaneously *B* grooms *A*, denoted *A* ↔ *B*). Regarding the duration of grooming events, we use the word “bout” to refer to an episode of dyadic grooming (where one or both individuals are grooming the other) with a clearly identifiable start and stop. For convenience, here we operationalized “bout” in a slightly different way from the cited studies, using the term to refer to an active, unbroken, grooming sequence where at least one of the two individuals is engaged in grooming (where the behaviour is either *A* → *B*, *A* ← *B*, or *A* ↔ *B*). Hence, here we use the term “within-bout” to mean grooming that happens within this unbroken sequence; and “between” bout to refer to any grooming that occurs after a break.

Previous studies have shown that within-bout time-matching is commonly observed in chimpanzees and other primates [28], [41], [49], [51]. We pursued the question of dyadic reciprocation after a delay (a delay referring to when grooming stops, and then restarts). Although using quite different methodology, we compare our results below to Gomes et al. [39], who studied a wild population of male and female adults at the Taï project (Côte d’Ivoire), and examined reciprocation amongst 91 dyads in three time frames: (1) within-bout, (2) within days, and (3) long term (across their entire dataset, up to 22 months). Using an index of reciprocation from 0 to 1 (higher values mean more time-matching), they found that within-dyad time-matching grew more symmetrical over longer time horizons (i.e. payback became more evenly time-matched). However, their analysis ignored a variable that we consider important: the precise length of time that it takes for reciprocation to occur. This is where our Delta scale fits in. To illustrate what we mean, imagine that *A* grooms *B*, then stops, and then, after a delay, *B* grooms *A*. If *B* started to groom *A* after some time period, which we denote Δ, after *A* had ceased grooming *B*, then, according to our “Delta Scale”, Δ is a positive number measured in units of time (which we will call an “inter-bout time delay”). Alternately, suppose that *B* starts grooming *A* at the precise instant that *A* ceases grooming *B*. This would be a Delta (Δ) of zero (no delay at all). If *B* started to groom *A* while *A* was in the midst of grooming *B* (causing *A* ↔ *B*), then Δ is negative (for more information about the Delta scale, see Methods). Gomes et al. [39], in contrast to our study, simply summed up the durations of the grooming events over different time periods (ignoring the difference between mutual grooming and delayed grooming). In contrast, our study below *explicitly* incorporates the delay in reciprocation as one of the variables in our model. This is a key innovation of our method.

Our methodology differed from Gomes et al. [39] in three additional ways. First, we sampled a captive population [49] instead of wild. Although this is a smaller project than Gomes et al. [39], we collected data on a very large number of grooming bouts (see Methods for details). Furthermore, at our field site, Chester Zoo, the sociability of the chimpanzees was very high compared to other zoos [52] and, therefore, our group generated a high volume of grooming data. Second, we grouped events in our dataset according to the *uninterrupted* period of time during which the investigator was present, and we deliberately *did not* aggregate data across these observation sessions. This was to avoid making incorrect inferences about time-matching over periods of time when data were absent. This led to fairly short windows allowing reciprocation (see Table 1), which here we call “short-to-medium-term” (we avoid referring to “long-term” is our study because other studies, e.g. ref. [39], had much longer time frames than we did). Finally, when summing grooming durations over a specified window, we compared our results against a null model in which grooming durations are independent and identically-distributed (*i.i.d.*) random variables. This allowed us to investigate the possibility that any time-matching effect that we observe is not simply due to the “law of large numbers” [53]. The law of large numbers is a statistical law, often poorly understood in everyday life [54], which describes how the value of a set of observations increases in accuracy as the sample size increases. In other words, the value (e.g. mean) of the observations becomes closer to the true value (e.g. the mean which would be obtained if all possible observations were made).

**Table 1:** The twenty-six observational sessions used in this study.

No.	Date	Start time	End time	Duration (minutes)
1	Nov 4	9:52	11:26	94
2	Nov 4	11:50	14:26	156
3	Nov 5	10:43	12:41	118
4	Nov 5	13:06	15:40	154
5	Nov 6	10:18	14:24	246
6	Nov 6	14:50	16:06	76
7	Nov 7	10:45	13:30	165
8	Nov 7	14:04	16:06	122
9	Nov 11	10:42	14:30	228
10	Nov 11	15:15	16:10	55
11	Nov 12	10:20	13:37	197
12	Nov 12	13:59	14:14	15
13	Nov 12	14:30	16:05	95
14	Nov 13	13:40	15:26	106
15	Nov 14	9:55	11:15	80
16	Nov 14	11:48	12:45	57
17	Nov 16	13:05	14:05	60
18	Nov 19	10:11	15:30	319
19	Nov 21	10:23	13:46	203
20	Nov 21	13:58	14:14	16
21	Nov 21	14:28	15:57	89
22	Nov 26	10:30	10:57	27
23	Nov 26	11:40	12:50	70
24	Nov 26	13:12	15:53	185
25	Nov 27	10:25	11:20	55
26	Nov 27	11:42	15:37	245

## Methods

Observational data were collected in November 2003 [49] on chimpanzees (*Pan troglodytes*, mixed subspecies) that were housed at Chester Zoo, UK, in a single group of 26 members (excluding infants) in an enclosure with an indoor and outdoor area [49], [55]. The chimpanzees typically spent the day in areas which were visible to the public (with the doors to the night cages usually not opened until late in the day). The public area of the indoor enclosure was inside a circular building with a cone-shaped roof. The indoor enclosure was 13m in diameter and 12m high [55]. The outdoor enclosure was 2000*m*^2^ [55] and connected to the indoor section by a single small door which was usually open. Except when the night cages were open, all individuals were usually visible to the public, sometimes at fairly close proximity (e.g. across a glass window), but other times at a longer distance (e.g. when outside). There were five adult males, fifteen adult females, four subadults, and two juveniles. The mean age at beginning of study was 18.7 years (SD = 11.4). All ages were included in the analysis (the youngest juvenile included in the data was 2.5 years old, at an age where chimpanzees start to reciprocate grooming [56]; see ref. [49] for specific information on individual grooming profiles for different ages of groomers). Most grooming occurred in the afternoon, just prior to the opening of their night cages. No data on dominance were collected. Kin relationships were partly known (see ref. [49], p. 270) from information provided by keepers. The research project was approved in advance by the research office at Chester Zoo.

### Data collection procedure

We analyzed 156.6 hours of grooming, but recorded within an overall period of 44.25 hours (spanning 17 days) over 26 separate observation sessions (see Table 1). As mentioned earlier, each measure of time-matching was restricted to within an observation session (e.g. looking at Table 1, if *A* → *B* occurred during the 9:26–11:26 session, then the reciprocation *B* → *A* was counted if it occurred before 11:26, but *not* counted if *B* → *A* occurred during the later 11:50–14:26 session). This session-restricted analysis had a deliberate point; if we were to pair events over periods of time when the observer was not present, then we could make erroneous inferences about time matching since events at the end of one session could have been reciprocated while the observer was absent (e.g. if the observer were present when *A* → *B* occurs, but then absent when *B* → *A* occurs). We return to this topic in the Discussion.

**Fig 1:**
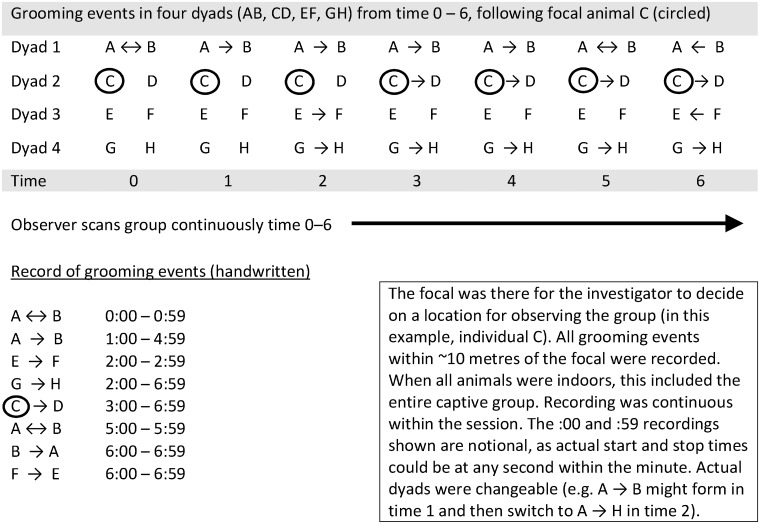
Schematic illustration of the data collection method, consisting of continuous all-occurrence sampling of grooming events within vicinity of a designated focal animal (for scan of actual datasheet, see ref. [49], p. 32). The original reason for designing the data sheet this way was to record the occurrence of polyadic grooming cliques: chimpanzees often groom within larger gatherings of individuals, which causes clusters of polyadic grooming [49], [57], such as triads (e.g. *A* → *B* ↔ *C*), quartets (e.g. *A* → *B* ↔ *C* ← *D*), or larger. Polyadic grooming is not the topic of the current paper, but the data sheets were usable for the analysis of dyadic grooming.

The procedure consisted of the investigator (Y.I.R.) continuously scanning a chosen group and recording all individuals and grooming cliques (identity of groomers, direction of grooming). As this was a novel methodology, this procedure is illustrated in Fig 1. Groups would often split apart and form separate grooming clusters, sometimes moving out of sight of other individuals that the observer had been recording. In order to decide to which groups to stay near, the observer chose a focal individual that he would follow throughout the session, and recorded each grooming episode within 10m of the focal individual (this means that recording would get censored for grooming bouts from which the focal animal walked more than 10m away). As this sample was collected during winter (November), the group were most often indoors and all together within the same indoor space.

Grooming events were timed to the nearest observed second using a single stop-watch. The investigator recorded the time that grooming started, the identity of the groomer(s), the recipient(s) of grooming, and the time that the grooming finished. In a previous publication on the same set of observations [50], but with differing measurements, an inter-observer reliability score for this method was calculated as 84.86%. For analysis, original handwritten data were transcribed into a text file (for all data files, see S1 Supplementary Material).

Recording grooming events to the nearest second without using a video camera has been done before (e.g. ref. [28]). Recording to the second required the focused concentration of the observer, particularly in moments where there were multiple grooming events simultaneously (see Fig 1). However, the Chester group was never observed to groom all at once. It was fairly uncommon to see more than three simultaneous grooming events. Obviously, despite the observer’s best efforts, the possibility of being accurate to the second might have been reduced when the observer became distracted or was trying to keep track of multiple events (e.g. being a few seconds late to notice that a chimpanzee had switched partners). Given the sheer amount of seconds of grooming we recorded, these slight uncertainties in recording the beginnings and ends of grooming events are unlikely to significantly affect our results.

### Data analysis procedures

We first analyzed our data by pairing overlapping or consecutive grooming events for every actively grooming dyad where grooming roles are reversed (reciprocated). For every such pair of events within the same observation session that relate to a dyad {*A*, *B*} we record:

- *X*—the duration that *A* groomed *B*;
- *Y*—the duration for which *B* groomed *A*; and
- Δ—the time between the end of the first event and the start of the next one.

**Fig 2:**
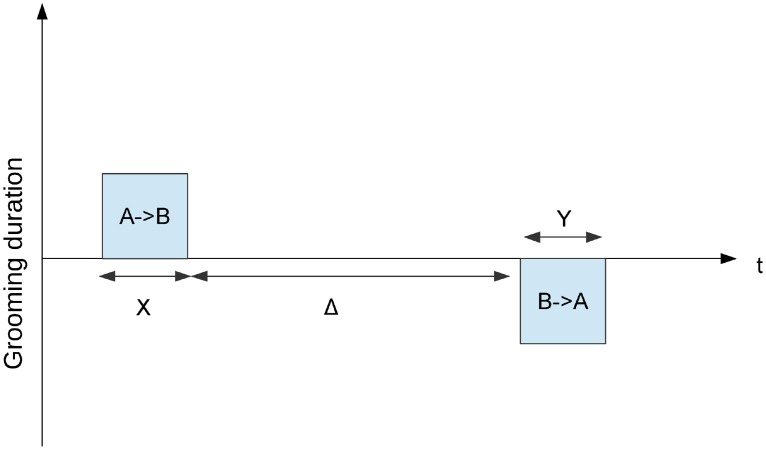
The model used for the longitudinal regression analysis. Consecutive or simultaneous pairs of grooming events between any given dyad {*A*, *B*} are represented in our dataset by triples (*X*, *Y*, Δ), where: *X* represents the time that *A* spent grooming *B*; *Y* represents the time that *B* spent grooming *A* and Δ represents the time that elapsed between (i) *A* finishing grooming *B*, and (ii) *B* starting to groom *A*. Within-bout grooming is recorded using negative values of Δ.

These variables are summarized graphically in Fig 2. Note that if the grooming events are simultaneous (i.e. within-bout grooming occurs, *A* ↔ *B*), then Δ can be negative. This is a crucial feature of our analysis as we can distinguish between within-bout reciprocation (*A* ↔ *B*), i.e. Δ < 0s, as compared with delayed reciprocation Δ ≥ 0s (*A* → *B*, then *A* ← *B*). Moreover, we can also analyze the distribution of the inter-bout time intervals.

We can understand how Fig 2 works by imagining that the two bouts (*X* and *Y*, i.e. *A* → *B* and *B* → *A*) can slide from left to right. As it appears on Fig 2 now, Delta is positive, Δ ≥ 0 (note the fairly big gap labelled Δ). Hence, Fig 2 is showing A grooming B (*X*), followed by a “Δ-length” gap, and then B grooming A (*Y*). To visualize a negative Delta, Δ < 0, imagine that the two bouts (*X* and *Y*) have slid over to be one on top of the other, at least partly overlapping. Here, the Δ line would not be *between* *X* and *Y*, but instead covering the space where *X* and *Y* *overlap*. This overlapping indicates immediate reciprocation in mutual grooming (*A* ↔ *B*). Although our approach to handling our data (as shown in Fig 2) might occasionally lead to the splitting of grooming bouts that might be construed as an uninterrupted bout (e.g. in the unlikely event that Δ is exactly zero), it allows us to be precise when we investigate the inter-bout time interval as a *variable* (zero merely being a mid-point on our continuous scale). This approach also avoids possible arbitrariness in setting a predefined threshold for Δ in order for *X* and *Y* to be considered as two separate bouts.

We analyzed these paired events by first producing descriptive statistics of a reciprocity measure *ρ* (rho) = |*X* − *Y*|/(*X* + *Y*) for every pair of consecutive or overlapping events within a given dyad and observation session where grooming roles are reversed (reciprocated). We partitioned the data into two comparison groups: i) *delayed* Δ ≥ 0, and ii) *within-bout* Δ < 0. When *ρ* = 0 there is no difference in the durations of the paired grooming bouts, and we can conclude that there is time-matched reciprocation, whereas values of *ρ* > 0 indicate asymmetry in grooming durations and a corresponding lack of time-matching. For our exploratory data analysis, we used box plots to compare the distributions of *ρ* in the within-bout group and the delayed group. Because our dataset consists of multiple grooming observations from each individual and dyad, we also examined the distribution of *ρ* by individual and dyad in order to ascertain whether there are any idiosyncratic responses for particular individuals or dyads. To test the hypothesis that delayed grooming has a significant effect on reciprocity, we used linear mixed-models with comparison group as the main factor, and the dyad identity as a random effect. This form of model was chosen because it allows us to account for the fact that multiple observations of *ρ* from each dyad are not independent replicates, and thus avoids the problem of pseudoreplication [58].

Our main result was established by testing for a significant difference of mean reciprocity ($\bar{\rho }$) in each comparison group (delayed versus within-bout) using a one-way repeated-measures analysis of variance (ANOVA) test using the dyad as the random effect, and then within each comparison group, testing whether the median reciprocity for each dyad ($\tilde{\rho_{d}}$) is significantly above zero. In the latter analysis, the null hypothesis is that there *is* time-matched reciprocity (i.e. $\tilde{\rho_{d}}=0$), and the alternative hypothesis is that there is asymmetry in time-matching; i.e. that durations are *not* matched ($\tilde{\rho_{d}}>0$). We reject the null hypothesis if the lower 95% confidence interval for $\tilde{\rho_{d}}$ is strictly positive. For comparison with other studies, we also examined scatter plots of time invested against grooming received to see whether there is any symmetry in grooming durations, and using the lme4 library [59], we performed linear regressions of the mixed-model *Y* = *X* + (1|*d*), where *d* is the dyad number: i.e. we regress grooming reciprocated with grooming received as a fixed effect, and the dyad as a random effect on the intercept.

Finally, for comparison with Gomes et al. [39], we also summed the durations of grooming events that occurred within consecutive time windows of various sizes, and then use a linear regression model in order to test whether aggregate grooming time is balanced within in each dyad. However, we argue that there is a fundamental problem with this method because if we assume chimpanzees groom randomly we would also observe considerable aggregate time matching by virtue of the *law of large numbers* [53]. Therefore, we also introduce a null model in which each individual’s grooming durations are drawn independently from a distribution with the same mean as the empirical data, and we compare the windowed time-matching analysis against the analysis of the data from the null-model. We used the R language and software environment [60] to perform the statistical analysis. The code used for our analysis is freely available under a creative commons license [61].

## Results

In the dataset spanning time periods totalling 44.25 hours, *n* = 2520 grooming events were recorded for an overall duration equal to 179.5 chimpanzee-hours. Therefore, the number of hours of recorded chimpanzee grooming (chimpanzee-hours) are >3.5 times longer than the total number of hours spent observing. Chimpanzee-hours exceed observation hours because it was not uncommon to see multiple grooming events occurring simultaneously [49]. The notional example in Fig 1 shows eighteen minutes’ worth of allogrooming data (0.3 chimpanzee-hours) collected within seven minutes of observation. Hence, we have an exceptionally dense grooming dataset.

**Fig 3:**
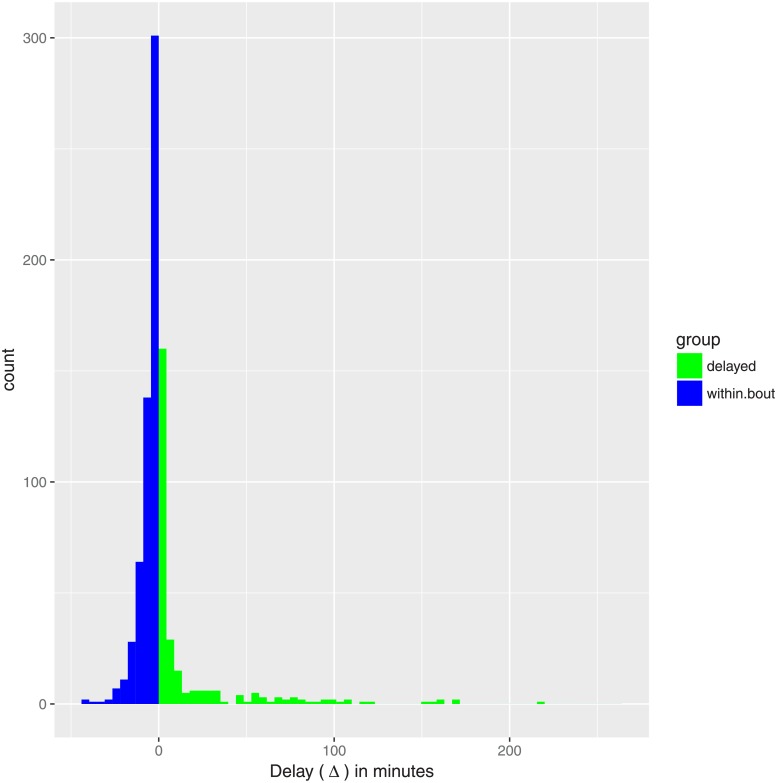
Histogram of Δ measured in minutes.

When we paired consecutive or simultaneous events where grooming roles were reversed, we were left with 156.6 chimpanzee-hours over a total of 832 *pairs* of grooming events, each associated with a particular dyad. Of these event pairs, 555 totalling 118.1 chimpanzee-hours (75%) consisted of within-bout grooming (Δ < 0), compared with 277 totalling 38.5 chimpanzee-hours (25%) for delayed grooming (Δ ≥ 0). The time delay Δ varied between −44 and 266 minutes (the full distribution is illustrated in the histogram in Fig 3). There were 146 actively grooming dyads observed (45% of a possible maximum of 325 dyads). Of the dyads observed, 137 (93.8%) showed mutual grooming at least once.

**Fig 4:**
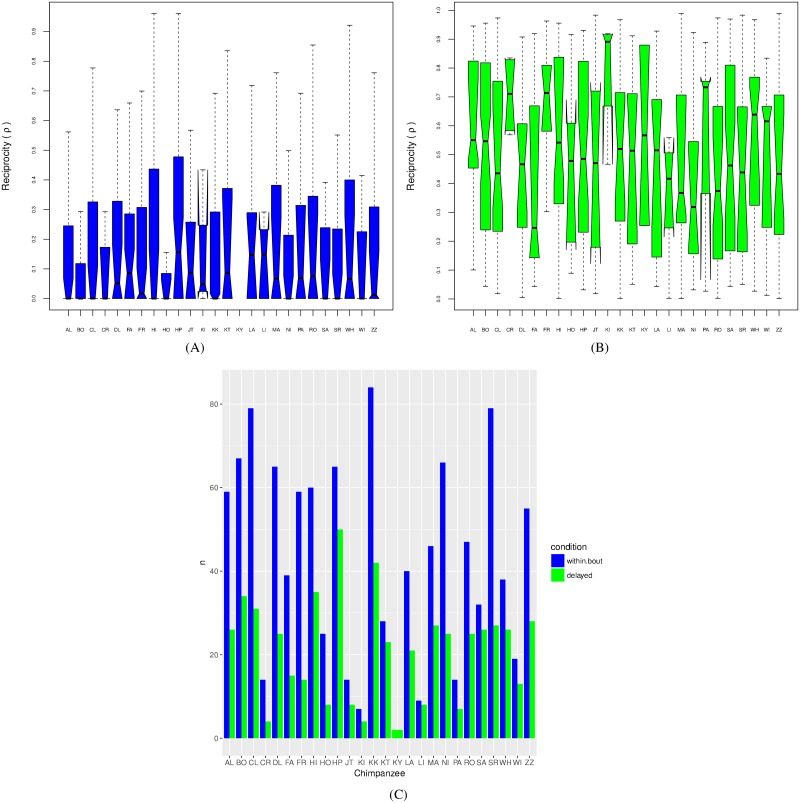
(A) Reciprocity scores (*ρ*_*i*_) for within-bout (Δ < 0) grooming by chimpanzee (*i*): one out of twenty six individual chimpanzees exhibit significant asymmetry in grooming urations ($\tilde{\rho_{i}}>0$) at *p* = 0.05; (B) Reciprocity scores for delayed (Δ ≥ 0) grooming: all individuals show significant asymmetry ($\tilde{\rho_{i}}>0$) at *p* = 0.05; (C) Corresponding sample sizes.

As mentioned in the Methods section, care needs to be taken in order to avoid pseudoreplication, and to avoid drawing conclusions from data which are dominated by observations from a minority of individuals. Hence, we first examine the reciprocity metric over individual chimpanzees and dyads. In Fig 4, we show the distribution of the reciprocity metric for each individual chimpanzee. Fig 4A shows the within-bout case Δ < 0, and Fig 4B shows the delayed case Δ ≥ 0. Corresponding sample sizes are summarized in Fig 4C. The notches in each boxplot show the 95% confidence intervals for the median, and we can reject the null hypothesis of reciprocal time matching ($\tilde{\rho }=0$) by testing whether the lower of these confidence intervals is strictly positive (i.e. > 0). In the delayed condition all individuals exhibit asymmetry in grooming durations ($\tilde{\rho }>0$), whereas in the within-bout condition only one individual out of 26 exhibits significant asymmetry in grooming durations (*p* = 0.05).

**Fig 5:**
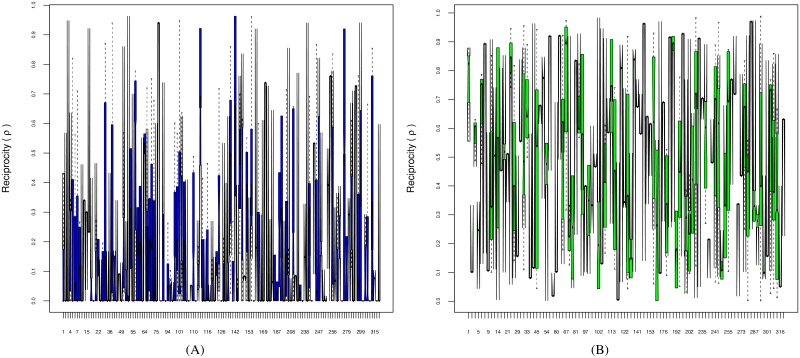
(A) Reciprocity scores (*ρ*_*d*_) for within-bout (Δ < 0) grooming by dyad (*d*): 32 out of 128 dyads exhibit significant asymmetry in grooming durations ($\tilde{\rho_{d}}>0$) at *p* = 0.05; (B) Reciprocity scores (*ρ*_*d*_) for delayed (Δ ≥ 0) grooming: all dyads show significant asymmetry ($\tilde{\rho_{d}}>0$) at *p* = 0.05.

Fig 5A and 5B show the same analysis for dyads for within-bout Δ < 0 and delayed Δ ≥ 0 respectively. In the delayed comparison group all dyads *d* exhibit significant asymmetry in grooming durations, i.e. median reciprocity $\tilde{\rho_{d}}>0$, whereas for the within-bout comparison group only 32/128 = 25% of dyads have significant asymmetry in grooming durations (*p* = 0.05).

Although median reciprocity is consistently above zero in the delayed comparison group, these analyses indicate indicate that there is still variation in reciprocity between individual chimpanzees and individual dyads, and clearly multiple observations from the same dyad cannot be considered independent replicates. Therefore in order to test whether delayed-grooming as a factor has significant effect on reciprocity we need to consider mixed models in which we allow for a random effect for each subject. Because our dataset consists of pairs of grooming events within each dyad, we consider dyads as subjects for the purposes of our statistical analysis (we provide a rationale for using dyads as subjects in our Discussion section).

**Fig 6:**
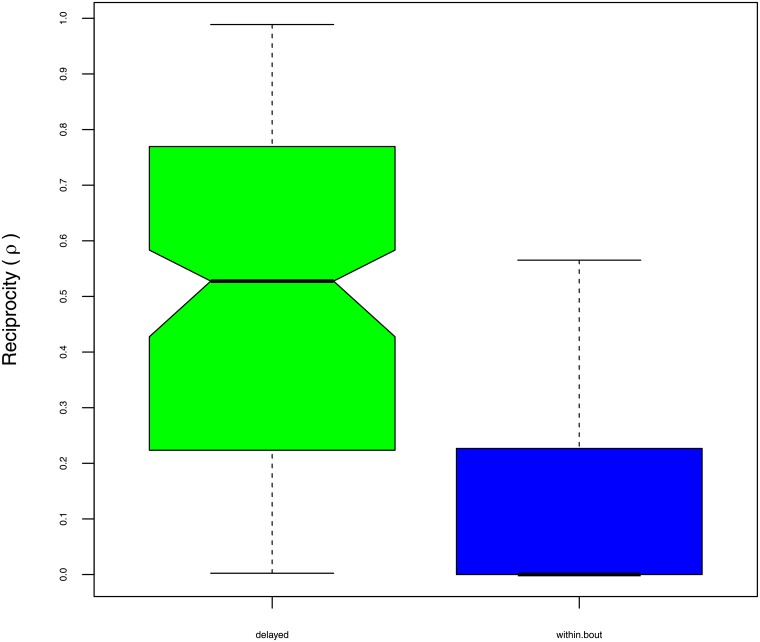
Box plots for the reciprocity measure *ρ* = |*X* − *Y*|/(*X* + *Y*) by comparison condition. Delayed grooming Δ ≥ 0 is shown on the left (*n* = 555), and within-bout grooming Δ < 0 on the right (*n* = 277).

A one-way repeated measures ANOVA was conducted to compare the effect of delay (Δ) on reciprocity (*ρ*) in the delayed (Δ ≥ 0) and within-bout (Δ < 0) conditions, using the dyad as a random effect. There was a significant effect of delay: F(1, 88) = 125.30, at *p* < 0.01. Moreover, the effect size was very large: *η*^2^ = 0.38, raw difference in means = 0.31, and Cohen’s *d* = 1.46. Fig 6 illustrates the overall effect size on the median values in each comparison group. All of the above results are consistent with the hypothesis that time-matching occurs within bouts (i.e. *ρ* = 0 when Δ < 0), but does not occur after a delay (*ρ* > 0 when Δ ≥ 0).

**Fig 7:**
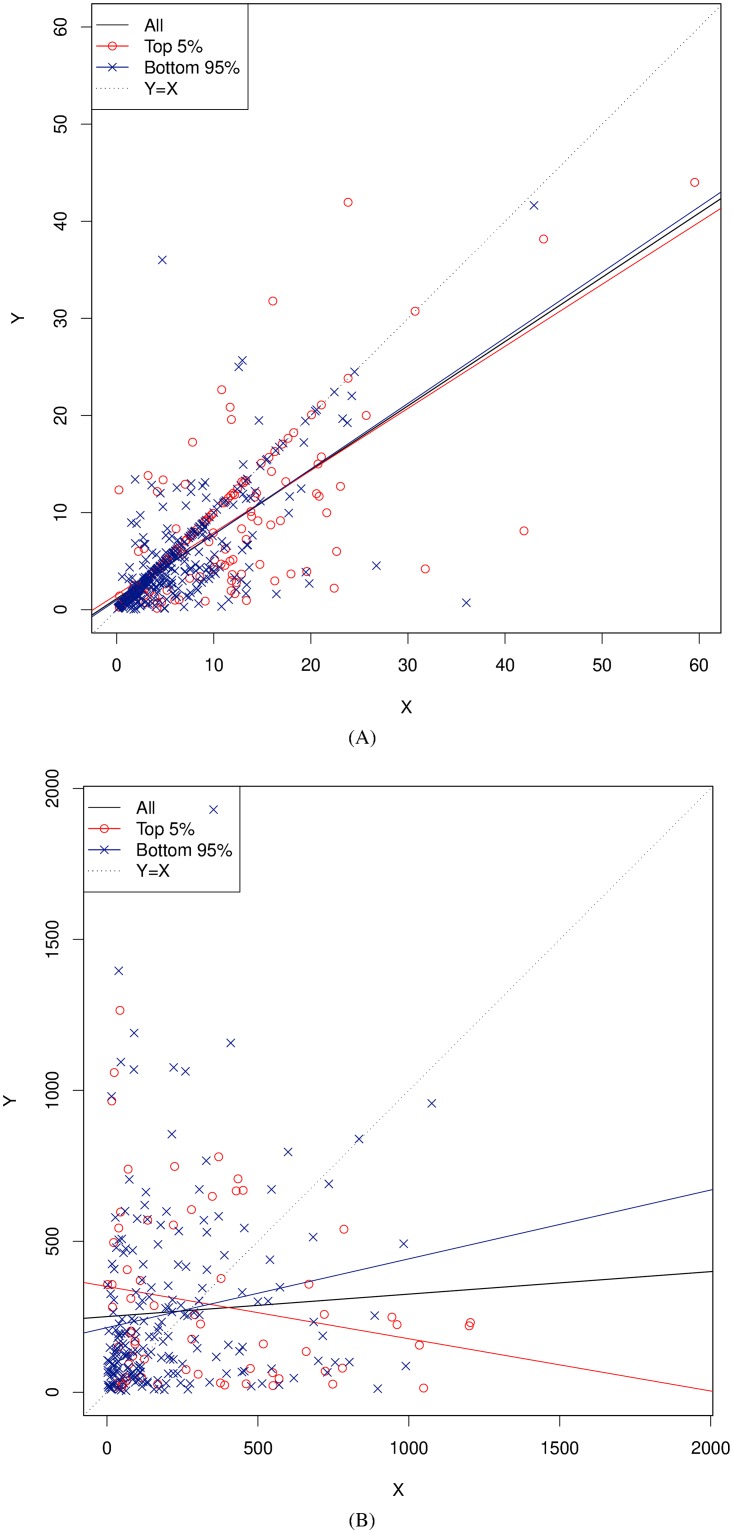
Time-matching in minutes, showing: (A) within-bout only and (B) delayed only. Here, *X* means grooming time invested (*A* → *B*) and *Y* means grooming reciprocated (*B* → *A*). Each point on the scatter-plots below represents a pair of grooming events for a single dyad {*A*, *B*}. The x-axis indicates the number of minutes that *A* spent grooming *B*, and the y-axis represents the time invested by *B* in grooming *A* (see text for further description of each panel).

**Fig 8:**
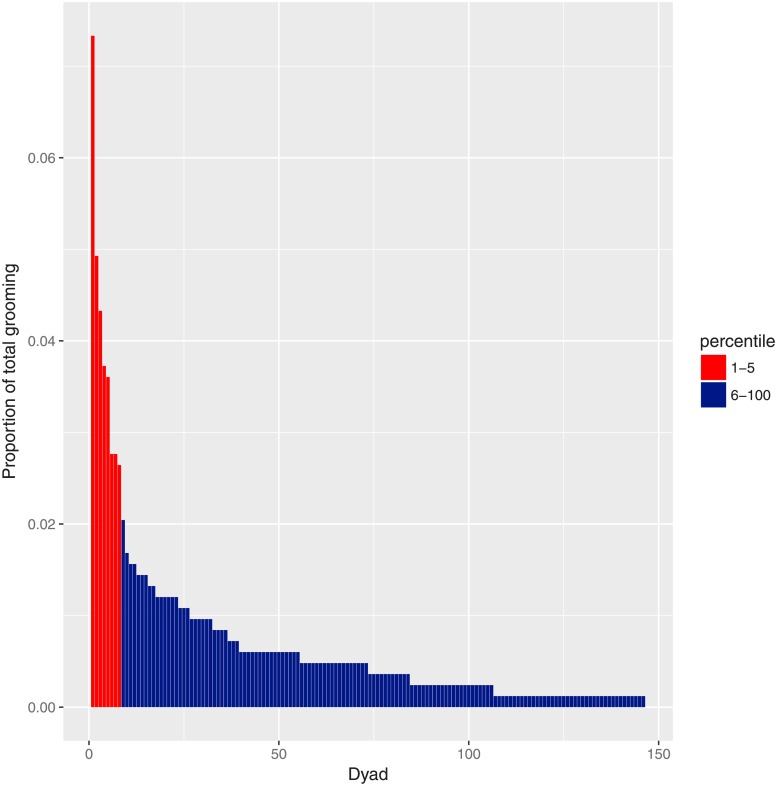
Distribution of total grooming duration over dyads. The dyads in the top five percentiles ({*AL*, *WH*}, {*BO*, *RO*}, {*DL*, *SA*}, {*HI*, *HP*}, {*KI*, *SR*}, {*KK*, *NI*}, {*KY*, *SA*}, {*SA*, *WI*}) are highlighted in red.

For comparison with studies, such as ref. [28], which use linear regression to test for time-matching, we directly examine the relationship between *X* and *Y* in the data, and look for symmetry by comparing with the model *Y* = *X*. Fig 7 illustrates the time-matching between dyads by plotting time invested (*X*) axis against time received (*Y*). Each point on the scatter-plots represents a pair of grooming events for a single dyad {*A*, *B*}; the x-axis represents the number of minutes that *A* spent grooming *B*, and the y-axis represents the time invested by *B* in grooming *A*. In order to detect possible over-representation (dyads who groom more frequently showing differing results from dyads that groom less frequently), we examine the distribution of the proportion of total grooming duration by dyad (Fig 8), and colour each pair of events according to whether it originated from a dyad in the top-5 grooming percentiles (red), or bottom 95 percentiles (dark blue).

The regression lines in Fig 7 show the O.L.S. (Ordinary Least Squares) estimates for the linear model *Y* ∼ *X*. Note that this regression is performed for illustration only; in Table 2 we use a mixed-effects model *Y* ∼ *X* + (1|*d*) to account for the fact that the observations for a given dyad are not independent. Fig 7A shows the data for the within-bout comparison group (Δ < 0), whereas Fig 7B shows delayed grooming (Δ ≥ 0). In the case of within-bout grooming, Fig 7A exhibits considerable clustering of data around the line *Y* = *X* indicating a considerable level of time-matching, and it is clear that cases where exact time-matching occurs are not dominated by the top groomers. However, the delayed case in Fig 7B shows no evidence of time-matching.

**Table 2:** Time-matching regression statistics obtained using the model *Y* ∼ *X* + (1|*d*), where *d* is the dyad number (i.e. we use a mixed-model with the dyad as a random effect on the intercept). The values in parentheses show standard errors of the estimates.

*Comparison group*	*α*	*β*	*p*-value
**within-bout**	68.43 (15.59)	0.66 (0.03)	< 2 × 10^−16^
**delayed**	246.10 (25.38)	0.049 (0.08)	0.47

In order to avoid pseudoreplication, we perform a time-matching regression using a mixed-effects model with the dyad as a random-effect on the intercept. Table 2 shows the mixed-model estimates and standard errors. The *p*-values were estimated using a likelihood ratio test with a null model comprising only the random effects. Once again, the analysis provides strong support for within-bout time-matching, but not in delayed time-matching. On the topic of kinship, when we incorporate the kinship of a dyad as a fixed-effect treatment into these analyses, we find that kin has no statistically-significant effect on the time-matching coefficients, nor does it improve the explained variance when incorporated as an additional variable in our regressions. We also found no effects when adding age as a fixed effect.

In the above analyses there is no evidence that dyads are time-matching their *consecutive* grooming events when these are separated by a delay. However, there still remains the possibility that if we aggregate grooming durations, for example by summing the grooming duration for each individual over consecutive time windows of a specified duration, then we might observe symmetry in the summed durations; this was the approach taken by Gomes et al. [39] when examining time-matching over different time horizons. However, because of the law of large numbers [53], great care needs to be taken in the design of a statistical test to detect such aggregate time-matching; if we sum two different samples *X* and *Y* each of which contain an equal number *n* of independently and identically-distributed random variates, then the corresponding sums can be made to equal one another with an arbitrary degree of precision simply by taking a sufficiently large sample (i.e. as *n* → ∞ we obtain $\bar{X}=\bar{Y}=\sum Y/n=\sum X/n$, and therefore ∑*Y* = ∑*X*). That is, under the null hypothesis that animals groom each other for an i.i.d. random duration, we will also observe at least some time-matching when durations are summed, and therefore we need to carefully distinguish between: i) the null hypothesis that individuals groom *randomly*, compared with (ii) the alternative hypothesis that individuals condition their reciprocal grooming with an equal *aggregate* duration within a given window of time.

For systematic comparison with Gomes et al. [39], we replicated their method of summing grooming durations over a specified time window, but crucially, in addition, we compare our results against a *null* model which assumes i.i.d. random grooming durations for each individual. Based on the length of our observation sessions, we examined four different time-horizons: 20 minutes, 40 minutes, 1 hour and 4 hours. For each time-horizon we partitioned the data into windows of the specified duration, and for each dyad, observation session and window we summed the grooming durations, plotting log(∑*X*) against log(∑*Y*) as in ref. [39].

**Fig 9:**
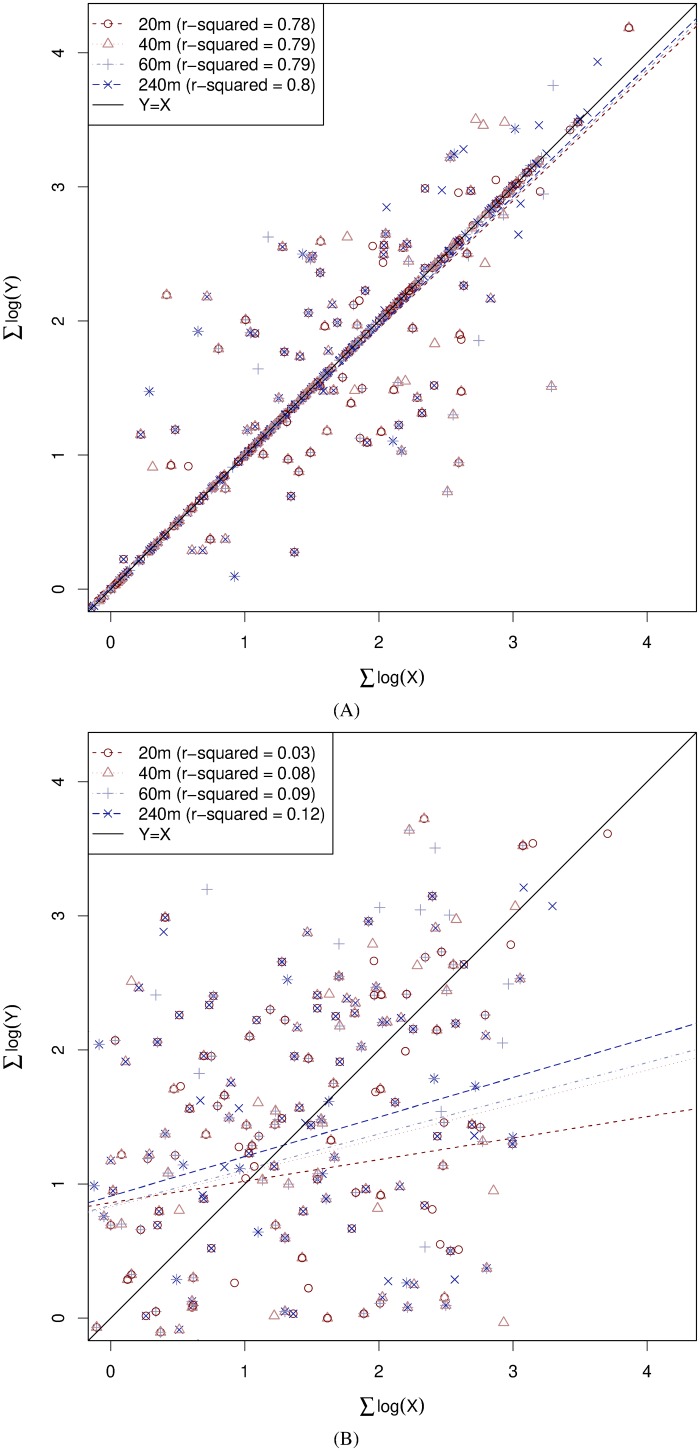
Windowed time-matching. The above plots illustrate time-matching when grooming durations are summed over time windows of 20 minutes, 40 minutes, 1 hour and 4 hours. The $\bar{R^{2}}$ values in parentheses in the caption beneath each figure shows the average of the *R*^2^ values over each regression within the comparison group. When we separate the data according to the delay Δ we see that most time-matching is accounted for by within-bout activity (A). When we restrict attention to delayed time-matching, the effect largely disappears (B).

This analysis is summarized in Fig 9. For each comparison group, we perform regressions of the model log(∑*Y*) ∼ log(∑*X*) for each window size, plotting the corresponding regression lines (dashed) in each subplot. All coefficients are significant at *p* < 0.05, but the fit of the model varies considerably, as summarized by the *R*^2^ values reported in Table 3. We see that windowed time-matching is only observed in the within-bout case (Fig 9A). When we restrict attention to the delayed case Δ ≥ 0 the symmetry disappears (Fig 9B).

**Table 3:** Sample sizes and adjusted *R*^2^ values for the regressions performed in Fig 9A and 9B.

Window size (minutes)	Comparison group	*n*	*R*^2^
20	**delayed**	246	0.03
40	**delayed**	232	0.08
60	**delayed**	224	0.09
240	**delayed**	203	0.12
20	**within-bout**	349	0.78
40	**within-bout**	323	0.79
60	**within-bout**	316	0.79
240	**within-bout**	288	0.79

All estimates were significant at *p* < 0.05.

Moreover, the fit of the model appears to increase as we increase the window size (Table 3). However, as discussed above, this could simply be due to the law of large numbers [53]; as we increase the window size, we increase the number of observations in the expression $\sum_{i=1}^{n}x_{i}=\sum_{i=1}^{n}y_{i}$, and even if the samples in each window, *X* = (*x*_1_, *x*_2_, …, *x*_*n*_) and *Y* = (*y*_1_, *y*_2_, …, *y*_*n*_), simply contain independently and identically drawn random values *x*_*i*_ and *y*_*i*_, we will obtain log(∑*Y*) = log(∑*X*) provided that the window size, and hence *n*, is sufficiently large.

In order to account for this, we compare the empirical analysis in Fig 9 against a null summation within time windows and regressions that we performed on the actual empirical data. To obtain data for the null model, the durations *X* and *Y* for every paired event in the original data are *replaced* with values drawn independently at random from the empirical distributions of grooming durations of the respective chimpanzees recorded in the original pair of events (in another words, for a particular pair of events pertaining to a particular dyad, the new null-model *X* and *Y* values are resampled with replacement from the entire cross-section of grooming durations of each of the chimpanzees in the dyad).

In order to quantify whether the aggregate time-matching results exhibit a statistically significant difference from the null model, we used a boot-strapping procedure to compare our results against those we expect under the null hypothesis of i.i.d. random grooming durations. We first compute the windowed version of our reciprocity metric *P*_*i*_ = |∑*X*_*i*_ − ∑*Y*_*i*_|/(∑*X*_*i*_ + ∑*Y*_*i*_) where *i* denotes the *i*^*th*^ time window containing durations *X*_*i*_ and durations *Y*_*i*_. We then compute this metric for all dyad-windows within each comparison group and for a given window size, in both the empirical data and in the data from the null model. We bootstrap by repeating this process for *n* = 10^5^ replications, using the median value of *P* computed across dyad-windows (denoted $\tilde{P}$) as our bootstrap estimator $\hat{P}$. We then take the 2.5 and 97.5 percentiles of resulting values as the critical values for $\tilde{P}$ at 95% confidence.

The results from bootstrapping are summarized in Table 4. The column with heading $\tilde{P}$ shows the median of windowed reciprocity for each comparison group, as calculated from the empirical data. The corresponding lower and upper critical values are obtained by bootstrapping from simulated data obtained under the null hypothesis. For the within-bout comparison group, the median windowed reciprocity is zero for all window sizes, and this value is well below the range of critical values at 95% confidence. However, in the case of delayed grooming we are unable to reject the null hypothesis of random i.i.d. grooming durations for each chimpanzee, irrespective of the window size.

**Table 4:** Median of the windowed reciprocity metric *P* computed across all window-session-dyads.

Window size (minutes)	Comparison group	$\tilde{P}$	lower ${\tilde{P}}_{crit}$	upper ${\tilde{P}}_{crit}$
20	**delayed**	0.48	(0.43)	(0.55)
40	**delayed**	0.45	(0.42)	(0.54)
60	**delayed**	0.44	(0.41)	(0.54)
240	**delayed**	0.41	(0.39)	(0.52)
20	**within-bout**	0.00	(0.44)	(0.55)
40	**within-bout**	0.00	(0.43)	(0.54)
60	**within-bout**	0.00	(0.42)	(0.53)
240	**within-bout**	0.00	(0.40)	(0.51)

This is compared against 95% lower and upper critical values obtained by bootstrapping from a null model in which grooming durations are independently and identically randomly-distributed for each individual. Lower values of *P* indicate superior time-matching when grooming durations are summed over the time windows of the specified duration. We used 10^5^ bootstrap replications. All results for the within-bout comparison group are significant at *p* < 0.05, however we are unable to reject the null hypothesis for delayed grooming.

Finally, we mention two alternate analyses (shown in S2 Supplementary Material, §S1–§S2). The first analysis (referring to Fig 2 in the main manuscript) arose from a reviewer comment that our choice of most-recent-*X* (instead of an earlier *X*) is an arbitrary decision. Accordingly, we ran an alternate analysis (shown in §S1) which produced equivalent results to those shown in the main manuscript. This new analysis did not change our conclusions. The second analysis also arose from a reviewer comment that time-matching will differ over different delay periods (e.g. how it differs after a few seconds delay versus an hour delay). We responded with an analysis shown in §S2. As shown there, that analysis did not produce significant results.

## Discussion

We had two starting questions: (1) to what extent do chimpanzees reciprocate grooming, and (2) over what time horizon? The answer to our first question (illustrated in Fig 7) is that reciprocation is considerably good when it happens concurrently. In other words, *A* tends to match B when in the midst of an allogrooming bout wherein *B* is grooming *A* (Δ < 0). This is a positive result. Reciprocation also exists in delayed grooming, but the time-matching is poor. In other words, after the allogrooming dyad ceases (Δ ≥ 0), the time-matching fidelity disappears (duration of *A* → *B* does not match up well with *B* → *A*). This is a negative result. The answer to our second question is that the time horizon is very short when it comes to accuracy in time-matching, but that reciprocation, in the short-to-medium term, does occur.

Hence, we obtained a part-positive, part-negative result. The most interesting part is neither the positive nor negative results in themselves but the disjuncture between these two results. Why should immediate reciprocity show excellent time-matching but delayed reciprocity show poor time-matching? Logically, our negative results do not allow us make a definitive statement that delayed time-matching does *not* occur. However, we did not find it here. There are various possible reasons for this. Below, we make four conjectures to explain our results.

### Four conjectures

Our first conjecture is that precise time-matching *does* exist but our data were not long-term enough for the matching to be complete (our longest possible measured delay was 4 hours and 26 minutes). In our study, we deliberately chose to minimize the possibility of unrecorded reciprocations (e.g. *A* grooms *B*, then observer goes on a coffee break and misses observing that *B* grooms *A*) by analysing reciprocations only within unbroken observation sessions (those shown in Table 1). This was advantageous for avoiding distortions in dyadic reciprocation budgets (e.g. *A* is recorded grooming *B* for 60 seconds, then *B* grooms *A* for 90 seconds but is *unrecorded*, and then *A* is recorded grooming *B* for 30 seconds, giving us the wrong impression of an asymmetrical budget of 90:0 in the grooming relationship when in fact it was perfectly matched at 90:90). But, a drawback of analysing only unbroken samples is that it severely limits the time range for analyses of reciprocation. We chose to do it this way because we could not record the chimpanzees 24/7 (to catch every possible reciprocation); but we felt that completeness was important in our particular type of analysis (to measure precision in time-matching). Based on our results, we cannot logically state the time-matching does not occur over longer time-frames. However, it is *implausible* that time-matching should become *more* precise as time goes by. Therefore, we should find the same disjuncture that we found between immediate and delayed time-matching even in longer term studies. A grooming event that is reciprocated after a six month gap is unlikely to show greater precision in time-matching than a grooming event reciprocated after a shorter gap. That said, we acknowledge that our results are not longitudinal enough to directly conclude anything on the long-term past a few hours.

Our second conjecture to explain our results is that time-matching *does* exist in the short-to-medium-term, but not in the precise second-by-second time-matching that we searched for in our study. If this were true, then perhaps our results are due to the impossibility of an animal being able to mentally measure precise units of time and, even if it *were* possible, for the animal to remember how many units were owed and by whom. In other words, cognitive constraints could limit the precision even if there were indeed motivation to pay back precisely (cf. [7]). If time-matching does exist, then perhaps it is more in the form of “*i* then reciprocate by *j*” [1] instead of “*amount* of *i* then *amount* of *j*.” In other words, the existence of the reciprocation event matters more than the duration of it (cf. [62]). A lack of time-matching precision is plausible given that even humans are not precise in keeping track of favors, particularly when it involves close friendships (cf. [12]). Furthermore, thinking back to our Delta scale, we acknowledge that the continuousness of the scale has implications for cognition at different points on the scale. Recent events will be remembered more vividly. Grooming duration should be relatively easy to remember if the grooming event were five seconds ago, or sixty seconds ago—but the precision of memory will decrease as minutes and hours pass. With the passage of time, memory for grooming events will become subject to the processes of compression and recency [63].

Our third conjecture is that delayed time-matching does not exist at all: any imbalance is set to zero after some time has passed. In other words, the prior history of interactions is irrelevant to (or at least not cognitively salient in) a current grooming bout. If this conjecture were true, then allogrooming partner choice is cognitively undemanding. However, the animal would need a very short memory, or be indifferent towards conspecifics, or be indifferent towards grooming. This does not describe primates, a highly social order, characterized by their capacity for highly individualized relationships and intense social bonds, something that was important in the survival and evolution of the primate lineage [27], [33], [34], [64], [65]. Furthermore, numerous studies have shown the importance of individualized relationships in primate studies [17], [20], [28], [30], [35], [36], [37], [38], [39], [40], [41], [42], [43], [44], [48], [50], [66], a key part of a biological market. In our own data in this study, we see the evidence of partner choice (only 45% of all possible dyads occurred) which suggests that chimpanzees were choosing partners in a deliberate fashion.

Our fourth conjecture relates to the wider biological market surrounding the dyadic grooming interactions. In the study above, we deliberately chose a stripped-down analysis, investigating nothing but allogrooming reciprocity. Yet, we know that allogrooming interactions are only one strand in a chimpanzee social life which is rich and complex [66]: there will be multiple contingencies in the social environment which will bear (perhaps weightily) upon allogrooming reciprocity patterns. Four possible sources of influence, as shown in research, are: (1) bystander effects, (2) contingencies between grooming and dominance, (3) variations in social relationships, and (4) patterns of interchange (allogrooming exchanged for a different currency) [20], [28], [35], [37], [40], [41], [42], [43], [44], [48], [49], [50]. The latter influence (interchange) could partly explain our results above which differentiate immediate and delayed time-matching. During allogrooming, a recipient of grooming usually has little else to do but groom back (although there are three other options: do nothing, depart the bout, or start grooming a third individual within reach [49], [57]). As long as the recipient stays in the bout, then allogrooming is the main type of active payback possible. However, once there is a delay in the resumption of an allogrooming dyad, there will have been an opportunity for *non-grooming* types of payback to occur. Hence, it is possible that time-matching after a delay has been confounded by intervening variables. Hence, it might appear, as it did in our analysis, that delayed time-matching is poor, only because we did not take into account the distorting effect of other currencies. In other words, our fourth conjecture is that other variables (such as intervening payback in a different currency, such as food sharing, cf. [45]) might have significant influence *between* bouts but less influence *within* bouts. Pertaining to our study, this is believable given what we know about biological markets. As we focused exclusively on grooming in our study, we cannot evaluate the effect of other currencies on our results. However, we are unsure whether it is a convincing idea that intervening currencies could actually reduce delayed time-matching down to virtually zero, as it did in our results. All of our conjectures above (shortness of time window, impossibility of precision, irrelevance of imbalance, influence of other contingencies) all should be explored further in future studies of sociality.

### Allogrooming and cognition

Cooperative behaviour is based on an animal’s decision-making abilities, which arose in the cognitive evolution of that species, and thus cognition and behaviour are inseparable sides of cooperation [7]. Despite an ever growing literature of experimental and observational work, there is still much debate about the exact character of the cognitive underpinnings of primate prosociality [5], [13], [66]. Thinking of chimpanzees in particular, we know that they do possess a long-term, autobiographical, memory [67], so it should not be surprising that allogrooming episodes should be included in that memory. Actual contingent cooperation (where *B* groomed *A* *because* *A* groomed *B*) is not particularly easy to prove [7]. Schino and Aureli [17] propose a mechanism of “reciprocal mutualism”, where cooperative exchanges (such as in grooming reciprocity) do not incur a reimbursable cost (as it would in “reciprocal *altruism*”), and furthermore, do not have cognitive requirements which are demanding. Instead, an occurrence of reciprocal mutualism only requires that an animal has an emotionally-mediated memory of its various relationships [17]. Applying this proposal to our studies, we conjecture that chimpanzees might remember, in an emotionally-directed way, which social interactions were successful and which were aversive (and thence maximize their grooming receipt by choosing to groom those who tend to reciprocate). It is more plausible that reciprocity, *if* cognitively salient to an animal, is more of the attitudinal kind (mirroring another’s attitude in the moment) [68] rather than a precise strategy that takes account of all relevant variables (the latter being a strategy that is unlikely even in humans, cf. [69]). The pertinence of our our results in this paper to the issue of cognitive abilities (although indirect at best) is that we found a distinct lack of evidence for precise time-matching after a delay—something that, had we found it—would have required fairly precise mental calculation. Whatever the case regarding the lack of precision after a delay, we can much more easily understand our positive result: the impressive precision of *immediate* time matching. The cognitive requirement here is simple: you groom another only as long as that other grooms *you*, based on the easy task of monitoring the current behaviour of the other.

Although conjecturing about cognition is important and interesting, we should stress that, in the study of reciprocity, one might separate the *allocation* (e.g. how much was transferred between *A* to *B* and then *B* to *A*) from the *process* (e.g. a psychological motive of fairness) that caused that allocation to occur [1]. In other words, a scientist can focus on analyzing the quantitative patterns of gift-giving reciprocation without necessarily needing to study the difficult question of an animal’s internal motivation. Thinking of reciprocation as analyzable on these two different planes [1], we can affirm that we have found interesting patterns of allocation without being conclusive about the cognitive processes involved.

### An economic system

On a functional level, the grooming market is a naturally-occurring economic system [30], [70], whose quantifiable rules of currency exchange are likely outside the comprehension of the animal. Grooming is unusual when we consider its currency-like properties. Because units of grooming time have value and are mutually interchangeable, they can construed as a medium-of-exchange. Units of grooming time have a benefit for the recipient but it is unclear what the costs are [25], [32]. Cost-free giving is not a foreign concept in economics (think of a country with the ability to sell assets such as natural resources which cost little or nothing to produce), but it does remind us not to draw too close an analogy between units of money and units of social grooming.

Our results appear incongruent with assumptions arising from the results of papers such as Gomes et al. [39] who showed that time-matching is occurring over a long time span. We know full well that our methodology is quite different, but the two studies can still be compared across conclusions that arise from the data. Accordingly, we are unsure about how their results square against ours: we showed time-matching ending after the bout ends and they showed time-matching over a very long time span. If our results are veridical, then it is possible that *their* results are not showing true reciprocity (sensu [1]), and instead their long-term time-matching results are due to the *law of large numbers* [53]. Applied to our time-matching data, the law of large numbers might come into play with a large sample size of grooming one-way (*A* → *B*) and grooming the other way (*B* → *A*). Assuming that the members of a given dyad tend to groom each other equally, the commencement of a grooming event could be viewed as an anchor (starting point) for time-matching to occur, and—despite fluctuations in the fidelity of time-matching in single bouts—with an increasing sample size of bouts, the average payback would eventually match up in both directions (t_*A* → *B*_ ≈ t_*B* → *A*_). Alternately, if members of a given dyad habitually groomed each other *un*equally (e.g. *2*t_*A* → *B*_ ≈ t_*B* → *A*_), then an increasing sample size will eventually produce a value that is close to reflecting the true measure of asymmetrical time-matching in *that* dyad [53]. Moreover, when averaged out over all dyadic relationships in the population (a mix of equal and unequal relationships), the law of large numbers might also lead to the eventual appearance of time-matching at the group level. However, this is all not to diminish the importance of the Gomes et al. [39] study. They did find time-matching. We might construe the time-matching that *they* found as a mostly non-cognitive strategy, but which plays an important functional role. Perhaps it is *not* important that time-matching is neither calculated nor premeditated. The important part might be just that it happens. The times match up eventually. Even if long-term time-matching is just a consequence of the law of large numbers, the fact that eventually it becomes equally reciprocated (amongst animals who are not counting the seconds) is perhaps enough to maintain a bond.

Additionally, we should also not ignore a simpler, more obvious, explanation for the difference between our results of that of Gomes et al. [39]. It might be that their wild group and our captive group differed in ways that differentiates the observed patterns. Allogrooming in chimpanzees has been called “friendly behaviour during rest” (ref. [46], p. 56) and at Chester Zoo, being a captive group, the chimpanzees had ample time to rest, and therefore ample time to groom. Congruent with this idea is that the Chester Zoo group has high rates of social behaviour relative to other populations [52]. Because of this and other factors (group composition, population/troupe size, spatial area), the biological market in our captive population versus the wild population at Taï will be topologically different and therefore we might expect inter-population differences in the emergent character of reciprocation. Furthermore, we did not have their wealth of longitudinal data. However, as our approach to the data were quite different from theirs, a direct comparison vis-à-vis topological differences would need further investigation.

### Conclusions

We acknowledge the limitations of our study. We investigated only chimpanzees living in a single zoo population, at short time frames. Although we believe that our conclusions might be applicable to longer-term wild populations, we present no direct evidence to conclude that. Furthermore, although we believe that our conclusions might be applicable to various forms of reciprocity, here we solely studied the currency of grooming, something which is only one of many possible currencies in biological markets [29], [30]. Grooming is in a different form from other types of currency (e.g. grooming is delivered as tiny units of stimulation, whereas other currencies, such as food, are delivered in bigger and more discrete chunks). Therefore, our results may only hold for allogrooming (or allogrooming-like) currencies. Finally, we should mention that grooming is more than just units of time. By evaluating grooming value simply by the number of minutes given out and received, we are ignoring the possibility that some units of time of grooming might be more valuable than other units. For example, a minute of allogrooming might be more *politically* valuable to a recipient if being given out by a “valuable” individual (e.g. a dominant, and/or coalition partner), or more *hedonically* valuable (e.g. if the grooming technique from one individual creates more pleasure than the technique from another).

Another notable issue is that we used *dyads as subjects* in our statistical analysis. We are not the first animal study to consider dyads as subjects for the purposes of statistical analysis (e.g. see ref. [71]). We felt that the dyad is an appropriate unit of analysis, because time-matching is intrinsically a property of a dyad, not an individual (i.e. the score applies to both individuals). Analyzing dyads as subjects is one recommended method for *avoiding* statistical independence issues (see ref. [72], p. 56). Non-independence is an inescapable part of analyzing dyadic data *if* the unit of analysis is the individual: there are partner effects (one influences the other), mutual effects (they influence each other), and “common fate” (both influenced by an external factor) [72]. Analyzing the dyad by-passes these problems, and is allowable when both members of the dyad have the same score [72]. Thinking of allogrooming, dyads might be considered non-independent because one individual might be a member of many dyads (e.g. chimpanzee *A* participates in grooming dyads *A*-*B*, *A*-*C*, *A*-*D*, etc.). Conversely, you might say that each relationship is a unique pattern of interaction (*A*-*B* ≠ *A*-*C* ≠ *A*-*D*…) and not necessarily dependent (cf. [72]). An extreme position might be that no member of an observed group can ever be truly independent (everyone influences each other; everyone is influenced by the same stimuli in the environment).

We can also put our results into the context of other primates. Allogrooming occurs in many species across the primate order [18], [25], [27]. Yet, chimpanzees in particular show grooming patterns dissimilar in form from other primate species: in the way they very often engage in mutual simultaneous grooming (*A* ↔ *B*) and complex chains of grooming (e.g. *A* ↔ *B* ← *C*) [49], [57]. There must be something interesting in the fact, that whereas mutual simultaneous grooming is rare in many monkey species (like those studied in refs. [28] and [51]), it is extremely common in chimpanzees. These complex configurations of grooming clusters in chimpanzees (dyadic, polyadic) perhaps create space for reciprocity to flow differently amongst chimpanzees than in other primates (e.g. reciprocity can flow more freely if grooming is not restricted to dyadic encounters requiring turn taking); and also have implications for other issues such as vigilance (being involved in mutual grooming would mean that both members in the dyad *A* ↔ *B* are focused on each other instead of potential threats in the environment). Another important difference to consider, between chimpanzees and other primates, is the species-typical sociality. Across the primate order, social organization and social structure occurs in diverse forms [25], [64]. For example, Gelada baboons typically live within a stable four-tier social organization: (1) grooming coalitions exist within (2) harems, which exist within (3) clans, which exist within (4) bands [25], [27]. Allogrooming in monkey societies will be distributed differently than in chimpanzee societies, the latter of which show fission-fusion sociality [44], [48], [73]. In chimpanzee groups, fission-fusion sociality (flexible and ever-changing groupings) will bring an individual into contact with a higher diversity of potential grooming partners than in geladas. Some researchers [74] have argued that the cognitive demands are higher in an environment of flux (such as in fission-fusion groups), and accordingly, great apes such as chimpanzees (in contrast to most monkey species) should be more future-oriented in their social reasoning. Why, then, did we find no evidence of delayed time-matching in our chimpanzee study? Perhaps, time-matching is just not necessary. Social competence is about flexibly and optimally adapting to social information as it changes [75]. Maybe it shouldn’t matter that chimpanzees are not good at being too calculating and too precise in their future-oriented planning. Humans are not good at it either [69].

The central result in our study shows a kind of cliff edge: time-matching becomes vastly less precise at the very instant a grooming bout ends. After a delay, the precision in gone. Reciprocation in the natural world, therefore, is perhaps not so cognitively laden as we like to think it is (precise paybacks only exist in human societies with money transactions). Future studies, using our general approach, might investigate delayed time-matching at longer time frames, with various time windows, with various species, and with currencies *other than* allogrooming, to investigate whether the same cliff edge is found in any naturally-occurring animal economy (societies without ledgers and banknotes) where prosociality depends on delayed reciprocity.

## Supporting information

S1 Supplementary Material Data files. (ZIP)

S2 Supplementary Material (1) Alternative event-pairing results and (2) Reciprocity over different delay periods. (PDF)
