# Supplementary material for: Precise time-matching in chimpanzee allogrooming does not occur after a short delay
Source: PLoS One. 2018 Sep 11;13(9):e0201810. doi: 10.1371/journal.pone.0201810 (PMC6133271; doi:10.1371/journal.pone.0201810)
Supplement: S2 Supplementary Material — (PDF) [file pone.0201810.s002.pdf]

Supplementary material for "Precise time-matching in chimpanzee allogrooming does not occur after a short delay"

Steve Phelps[1], Wing Lon Ng[2], Mirco Musolesi[3] and Yvan I. Russell[4,5]*
[1]Department of Informatics, King's College London, London, UK.
[2]Independent Scholar, London, UK
(affiliated with Bounded-Rationality Advancement in Computational Intelligence Laboratory)
[3]Department of Geography, University College London, London, UK.
[4]Department of Psychology, Middlesex University, London, UK.
[5]CRC Evolution of Social Behaviour, University of Göttingen, Göttingen, Germany.
*Corresponding author: yvanrussell@gmail.com

August 10, 2018

§ S1. Alternative event-pairing results.

An interesting issue, mentioned by a reviewer, is that there is some arbitrariness in choosing the most recent event as the starting point for analysis. As shown in Fig 2 of the main manuscript, after X occurs ($A \rightarrow B$), then it is compared to the occurrence of Y ($B \rightarrow A$). Why not, for example, choose an X that happened earlier, such as two events earlier? In other words, if $X1$, then $X2$, occurred before Y, then why not choose $X1$ instead of $X2$? We agree that this is a valid point, and so we conducted an alternate analysis using $X1$ (an earlier event) instead of $X2$. In light of that reviewer's comment, we reran our analysis with this alternative pairing procedure. The results are presented below using plots in an identical format to the original manuscript, and the windowed time-matching results are summarised in S1 Table. As can be

Table S1: Median of the windowed reciprocity metric P computed across all window-dyads.

Window size (minutes)	Comparison group	$\widetilde{P}$	lower P_{crit}	upper P_{crit}
20	**delayed**	0.53	(0.46)	(0.60)
40	**delayed**	0.45	(0.45)	(0.59)
60	**delayed**	0.49	(0.45)	(0.59)
240	**delayed**	0.46	(0.44)	(0.58)
20	**within-bout**	0.00	(0.45)	(0.56)
40	**within-bout**	0.00	(0.43)	(0.55)
60	**within-bout**	0.00	(0.43)	(0.55)
240	**within-bout**	0.00	(0.42)	(0.54)

This is compared against 95% lower and upper critical values obtained by bootstrapping from a null model in which grooming durations are independently and identically randomly-distributed for each individual. Lower values of P indicate superior time-matching when grooming durations are summed over the time windows of the specified duration. We used 10^5 bootstrap replications. All results for the within-bout comparison group are significant at $p < 0.05$, however we are unable to reject the null hypothesis for delayed grooming.

seen, the alternative pairing procedure *does not* alter the main conclusion of our manuscript: time-matching does not occur after a delay.

The figures in §S1 all correspond to figures in the main manuscript. S1 Fig corresponds to Fig 3 in the main manuscript. S2 Fig corresponds to Fig 6 in the main manuscript. S3(a), S3(b), and S3(c) Figs correspond to Figs 4(a), 4(b), and 4(c) in the main manuscript, respectively. S4(a) and S4(b) Figs correspond to Figs 5(a) and 5(b) in the main manuscript, respectively. S5(a) and S5(b) Figs correspond to Figs 7(a) and 7(b) in the main manuscript, respectively. S6 Fig corresponds to Fig 8 in the main manuscript. S7(c) and S7(d) Figs correspond to Figs 9(a) and 9(b) in the main manuscript, respectively.

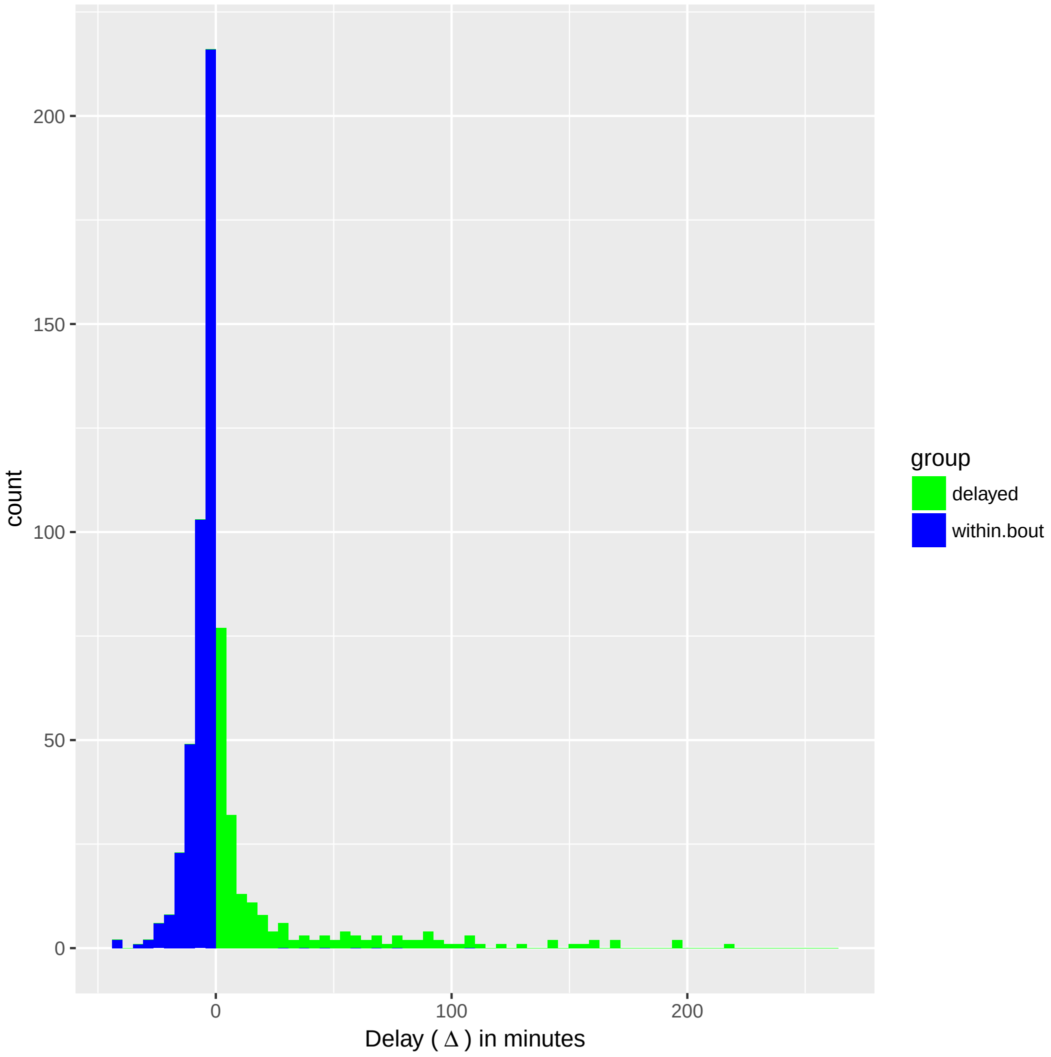

Figure S1: Histogram of Δ measured in minutes.

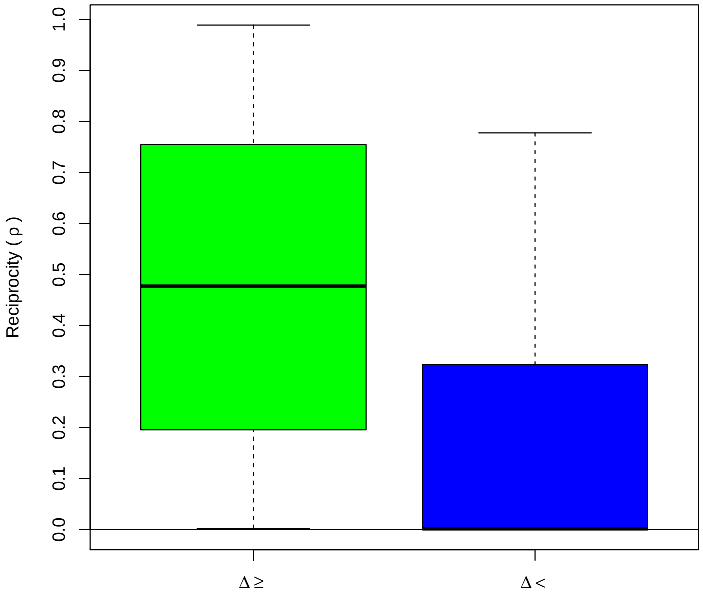

Figure S2: Box plots for the reciprocity measure $\rho = |X - Y|/(X + Y)$ by comparison condition. Delayed grooming $\Delta \geq 0$ is shown on the left ($n = 555$), and within-bout grooming $\Delta < 0$ on the right ($n = 277$).

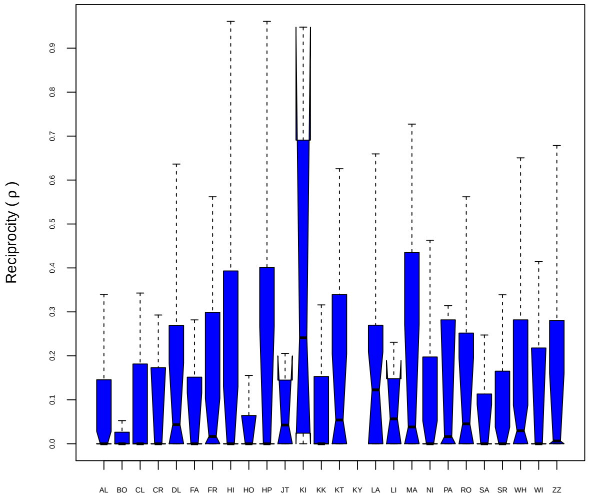

(a) Reciprocity by chimpanzee - $\Delta < 0$

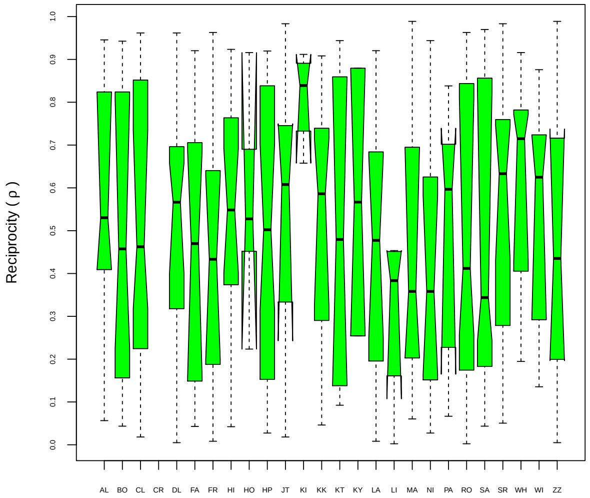

(b) Reciprocity by chimpanzee - $\Delta \geq 0$

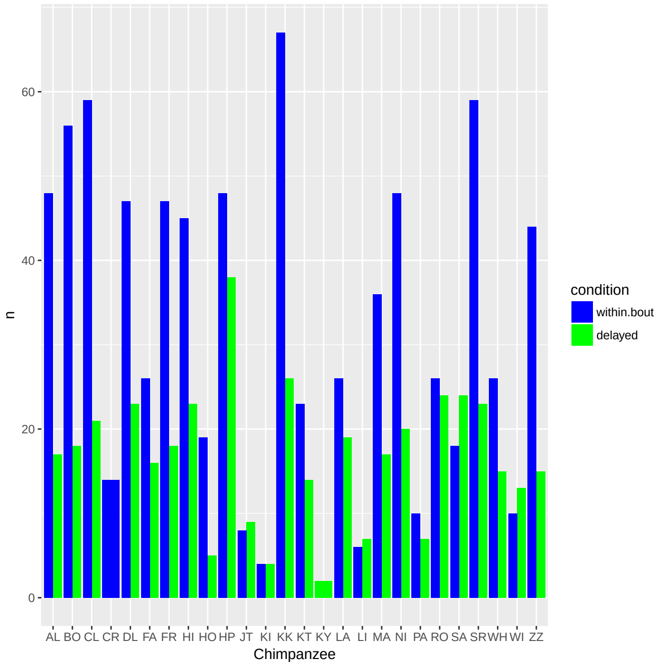

(c) Number of paired events by chimpanzee

Figure S3: Box plots for the reciprocity measure $\rho = |X - Y|/(X + Y)$ grouped by individuals. Fig (a) is restricted to within-bout grooming — i.e. $\Delta < 0$ — whereas (b) illustrates the delayed case $\Delta \geq 0$. The corresponding sample sizes are summarised underneath in (c).

.

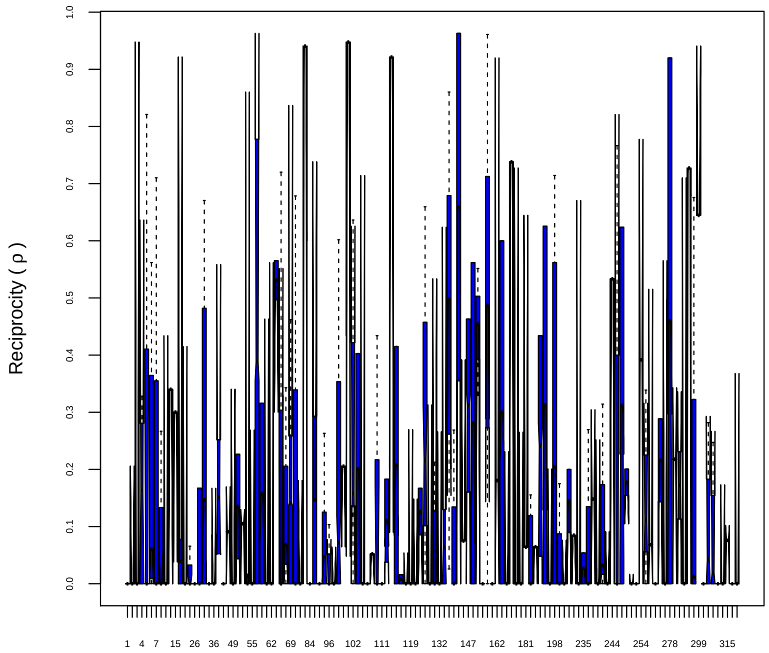

(a) Reciprocity by dyad - $\Delta < 0$

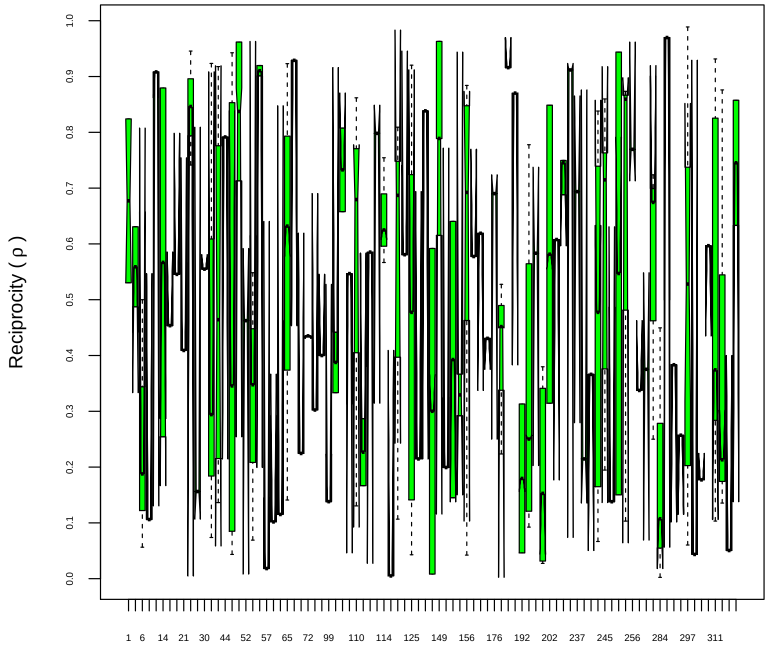

(b) Reciprocity by dyad - $\Delta \geq 0$

Figure S4: Box plots for the reciprocity measure ρ grouped by dyad. Fig (a) is restricted to within-bout grooming — i.e. $\Delta < 0$ — whereas (b) illustrates the delayed case $\Delta \geq 0$.

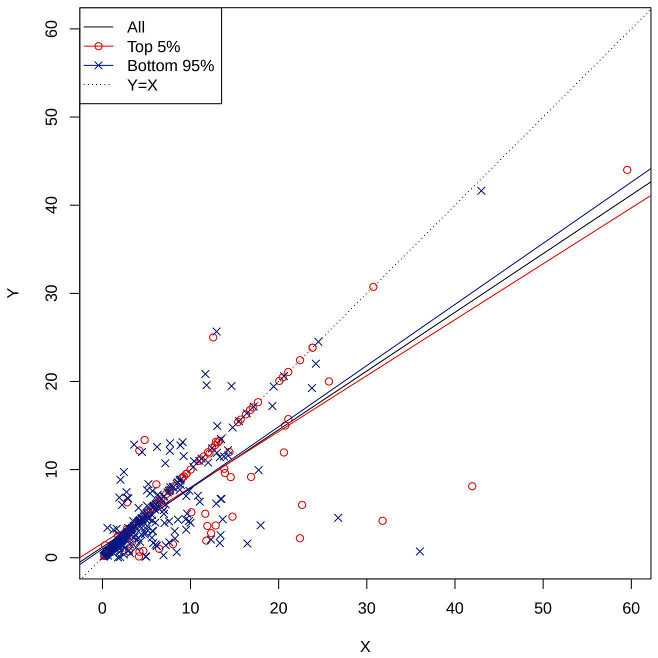

(a) Within-bout time-matching $\Delta < 0$

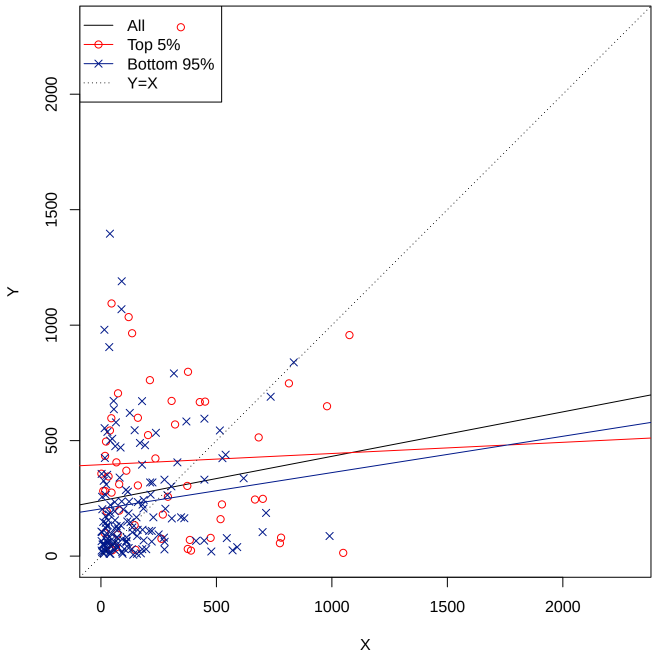

(b) Delayed time-matching $\Delta \geq 0$

Figure S5: Time-matching in minutes, showing: (a) within-bout only and (b) delayed only. Here, X means grooming time invested ($A \rightarrow B$) and Y means grooming reciprocated ($B \rightarrow A$). Each point on the scatter-plots below represents a pair of grooming events for a single dyad $\{A, B\}$. The x-axis indicates the number of minutes that A spent grooming B, and the y-axis represents the time invested by B in grooming A.

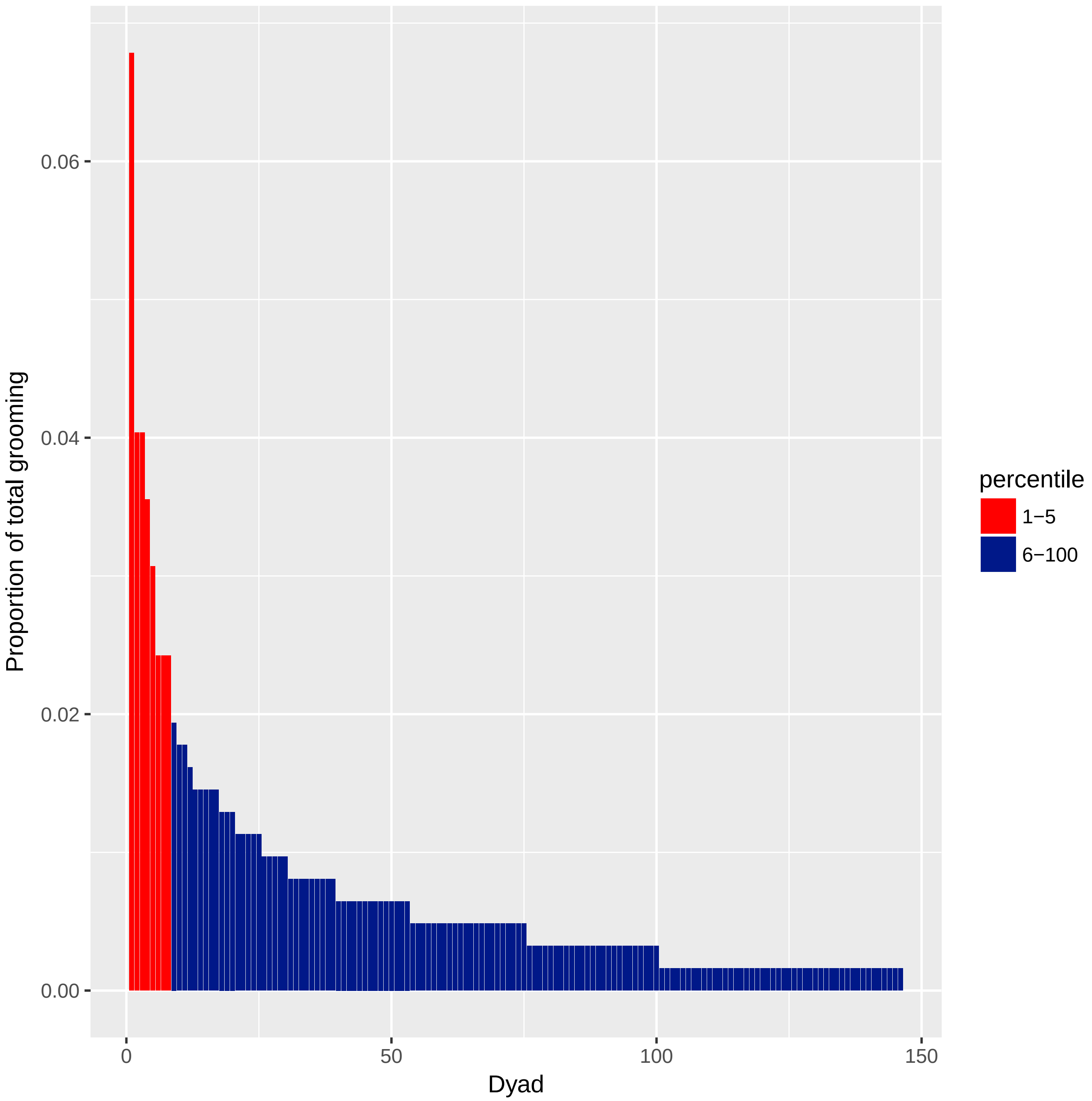

Figure S6: Distribution of total grooming duration over dyads. The dyads in the top five percentiles ($\{AL, WH\}$, $\{BO, RO\}$, $\{DL, SA\}$, $\{HI, HP\}$, $\{KI, SR\}$, $\{KK, NI\}$, $\{KY, SA\}$, $\{SA, WI\}$) are highlighted in red.

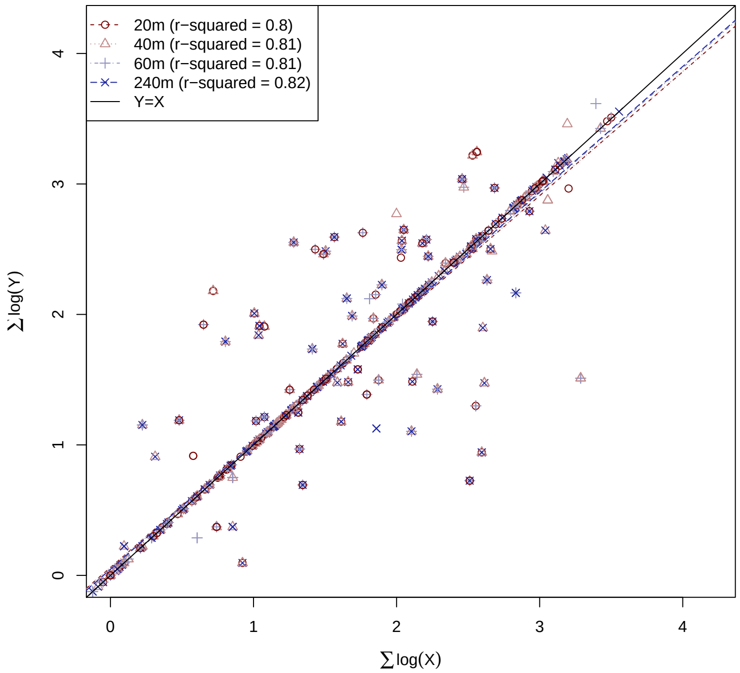

(a) Within-bout $\Delta < 0$ ($\bar{R^2} = 0.79$)

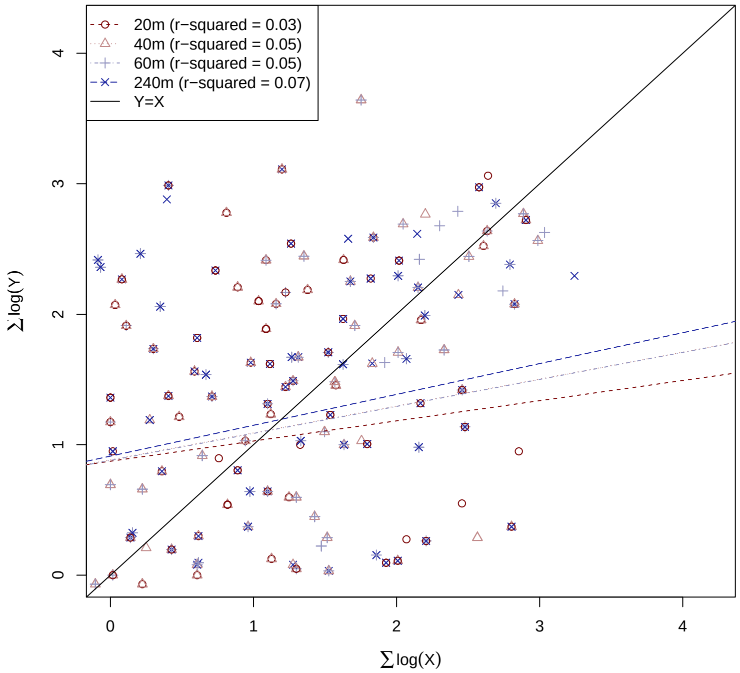

(b) Delayed $\Delta \geq 0$ ($\bar{R^2} = 0.08$)

Figure S7: Windowed time-matching. The above plots illustrate time-matching when grooming durations are summed over time windows of 20 minutes, 40 minutes, 1 hour and 4 hours. The $\bar{R}^2$ values in parentheses in the caption beneath each figure shows the average of the R^2 values over each regression within the comparison group. When we separate the data according to the delay Δ we see that most time-matching is accounted for by within-bout activity (a). When we restrict attention to delayed time-matching, the effect largely disappears (b).

§ S2. Reciprocity over different delay periods.

The same reviewer also suggested that although reciprocity might not occur after a very long delay due to memory decay, if we restrict attention to shorter delays, then we might find evidence of time-matching. We examined this question in a previous version of the manuscript, but excluded the analysis from the main manuscript. However, in light of the reviewer's comment, we present those results here. We used a 20-minute moving time-window over Δ, and performed time-matching regressions on data within the data, showing the regression slope (S8 Fig) and fit (S9 Fig). As can be seen, there is no evidence of time-matching once the window excludes data from $\Delta \leq 0$. The figures in §S2 are not corresponding to any of the figures in the main manuscript.

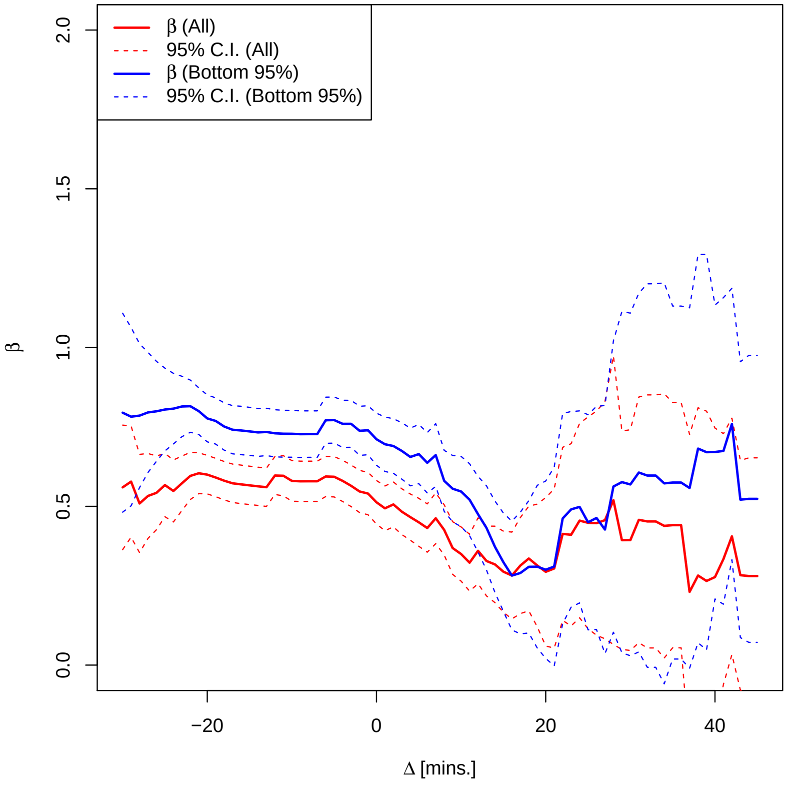

Figure S8: Time-matching regression results for a 20 minute moving window of Δ showing slope and associate confidence intervals as the delay changes. Results are not statistically significant once the window moves beyond the 0-20 minute period.

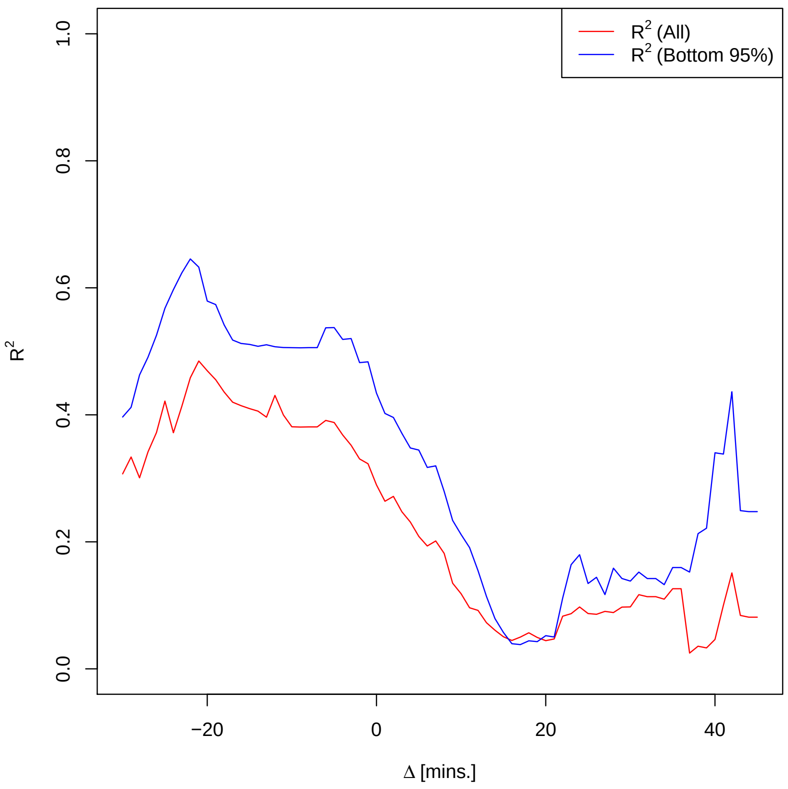

Figure S9: Time-matching regression results for a 20 minute moving window of Δ showing R^2. The fit becomes very poor once the window moves beyond the 0-20 minute period.
